# Steric Hindrance of NH_3_ Diffusion on Pt(111) by Co-Adsorbed O-Atoms

**DOI:** 10.1021/jacs.2c10458

**Published:** 2022-11-18

**Authors:** Dmitriy Borodin, Oihana Galparsoro, Igor Rahinov, Jan Fingerhut, Michael Schwarzer, Stefan Hörandl, Daniel J. Auerbach, Alexander Kandratsenka, Dirk Schwarzer, Theofanis N. Kitsopoulos, Alec M. Wodtke

**Affiliations:** † Institute for Physical Chemistry, Georg-August University of Goettingen, Tammannstraße 6, Goettingen37077, Germany; ‡ Department of Dynamics at Surfaces, Max Planck Institute for Multidisciplinary Sciences, Am Fassberg 11, Goettingen37077, Germany; § Donostia International Physics Center (DIPC), Paseo Manuel de Lardizabal 4, Donostia-San Sebastián20018, Spain; ∥ Kimika Fakultatea, Euskal Herriko Unibertsitatea UPV/EHU, P.K. 1072Donostia-San Sebastián20018, Spain; ⊥ Department of Natural Sciences, The Open University of Israel, Raanana4353701, Israel; # Department of Chemistry, University of Crete, Heraklion71500, Greece; ∇ Institute of Electronic Structure and Laser − FORTH, Heraklion70013, Greece; ○ International Center for Advanced Studies of Energy Conversion, Georg-August University of Goettingen, Tammannstraße 6, Goettingen37077, Germany

## Abstract

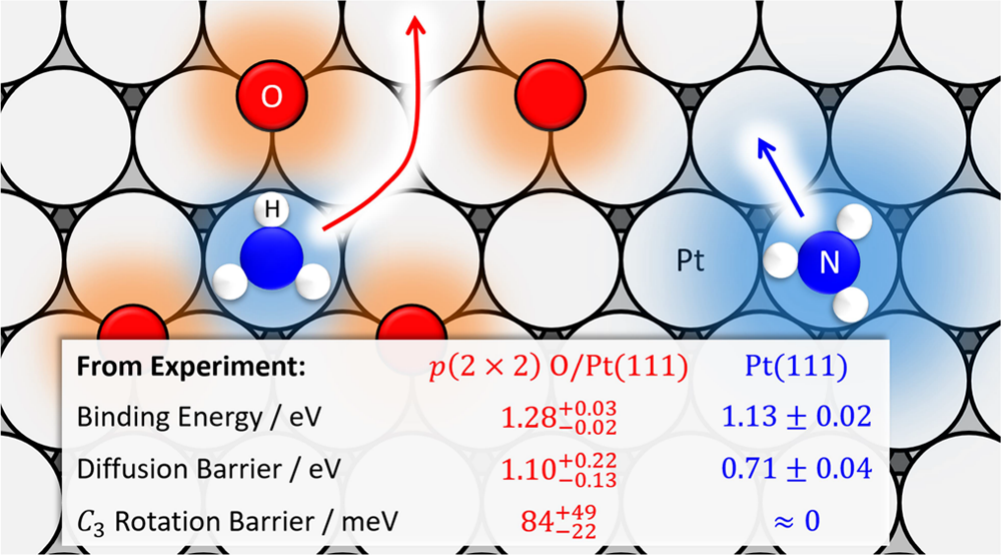

A detailed velocity-resolved kinetics study of NH_3_ thermal desorption rates from *p*(2 × 2) O/Pt(111) is presented. We find a large reduction in the NH_3_ desorption rate due to adsorption of O-atoms on Pt(111). A physical model describing the interactions between adsorbed NH_3_ and O-atoms explains these observations. By fitting the model to the derived desorption rate constants, we find an NH_3_ stabilization on *p*(2 × 2) O/Pt(111) of 0.147_–0.014_^+0.023^ eV compared to Pt(111) and a rotational barrier of 0.084_–0.022_^+0.049^ eV, which is not present on Pt(111). The model also quantitatively predicts the steric hindrance of NH_3_ diffusion on Pt(111) due to co-adsorbed O-atoms. The derived diffusion barrier of NH_3_ on *p*(2 × 2) O/Pt(111) is 1.10_–0.13_^+0.22^ eV, which is 0.39_–0.14_^+0.22^ eV higher than that on pristine Pt(111). We find that Perdew Burke Ernzerhof (PBE) and revised Perdew Burke Ernzerhof (RPBE) exchange–correlation functionals are unable to reproduce the experimentally observed NH_3_–O adsorbate–adsorbate interactions and NH_3_ binding energies at Pt(111) and *p*(2 × 2) O/Pt(111), which indicates the importance of dispersion interactions for both systems.

## 1 Introduction

The study of enzymes has provided us many of the prototypical concepts of catalysis, including the idea that a catalyst lowers the barrier to reaction and thereby accelerates the approach to chemical equilibrium. Enzymes are able to bind reagents to the active site directing them into an energetically stabilized structure that resembles the reaction’s transition state.^1−3^ This catalytic mechanism is the result of millions of years of evolution that produced steric effects, originally described by a “lock and key” model.^3^ These steric effects successfully accelerate reactions at the moderate temperatures of living beings. By contrast, reactions on man-made industrial catalysts using metal surfaces often deviate strongly from this elegant picture. Industrial catalysts typically operate at elevated temperatures enhancing the influence of desorption. Furthermore, there are no lock-and-key properties that bring the reactants to the active site. Instead, surface diffusion accomplishes this function; thus, a reactant’s ability to diffuse on the catalyst surface to the active site while competing against thermal desorption may determine the catalyst’s activity. This points out the importance of accurate determination of thermal diffusion coefficients and desorption rate constants under realistic reaction conditions on catalytic surfaces.

The high coverages produced under industrial high-pressure conditions often lead to complex steady-state surface structures involving interactions between different adsorbates.^5−8^ As a result, the potential energy landscape for diffusion of one reactant may depend strongly on the presence (or absence) of the other.^9,10^ Determining this energy landscape and how it depends on surface structure and adsorbate concentration represents a fascinating problem in physical chemistry of great relevance to understanding catalytic behavior. Unfortunately, disentangling the diffusional influences on catalytic activity from the conventional barrier-reducing effects can be quite challenging, since these normally occur in parallel. This is the reason why for many important catalytic reactions, little is known about the impact of co-adsorbate interactions on reactant and transition state energies and entropies. The situation is not made easier by the fact that state-of-the-art electronic structure theory—density functional theory (DFT) at the level of generalized gradient approximation (GGA)—is unable to describe van der Waals (vdW) interactions that are thought to be crucially important to a correct description of adsorbate–adsorbate interactions.^11−14^

Accurately measuring the kinetic competition of diffusion and desorption under industrially relevant high-temperature conditions has recently become possible with the introduction of the velocity-resolved kinetics (VRK) method.^15,16^ This method provides highly accurate desorption rate constants that can be modeled by statistical rate theories to yield adsorbate binding energies and entropies. Recent work showed that NH_3_ diffusion on Pt(111) is remarkably slow, due to an unexpectedly high diffusion barrier of 0.71 ± 0.04 eV constituting ∼65% of the NH_3_ binding energy to Pt.^17^ This called into question the wisdom of a generally applied assumption in the modeling of industrial catalysis that diffusion is not a rate-determining process.^9,18,19^ Within the context of NH_3_ oxidation on Pt, we hypothesized that NH_3_ diffusion could be further influenced by co-adsorbed oxygen atoms on Pt(111), which will be also present at the catalyst under industrial ammonia oxidation conditions.^17^

Here, we present a detailed VRK study of NH_3_ desorption and diffusion on *p*(2 × 2) O/Pt(111). We observe that when compared to Pt(111) at the same temperature, the thermal desorption rate is substantially reduced. In agreement with previous work, we find no evidence for NH_3_ decomposition or oxidation on Pt(111), which means that the observed differences in desorption rates are attributed to nonreactive interactions between NH_3_ and O. The temperature dependence of the desorption rate constants shows that NH_3_ binds more strongly to Pt(111) in the presence of co-adsorbed O-atoms and that the adsorbed NH_3_ entropy is substantially reduced. Using a noncovalent additive interaction model (NC-AIM) describing attractive and repulsive forces acting between NH_3_ and O on Pt(111), we could reproduce the observed thermal desorption rates quantitatively over a broad temperature range. In this way, the NC-AIM explains the steric interactions between adsorbed O-atoms and NH_3_ molecules. Specifically, NH_3_ exhibits a barrier of 0.084_–0.022_^+0.049^ eV for rotation about its N–Pt bond (no barrier on Pt(111)), an increased diffusion barrier of 1.10_–0.14_^+0.22^ (0.71 ± 0.04 eV on Pt(111)), and an increased binding energy 1.28_–0.02_^+0.03^ eV (1.13 ± 0.02 on Pt(111)). These results provide critical benchmarks for first-principles calculations of nonreactive interactions between NH_3_ and O on Pt(111). Comparing these quantities to DFT predictions reveals that dispersion corrections are crucial for obtaining a chemically accurate description of the energetic landscape. These findings set a strong basis for a comprehensive kinetic modeling study of ammonia oxidation for the Ostwald process.

## 2 Results

The methods used in this work are described in detail in Section 5.1. Briefly, the surface is dosed with a short molecular beam pulse of NH_3_ seeded in He (1%, 5 × 10^–4^ ML/pulse) and the flux of thermally desorbing molecules is obtained using spatial ion imaging. The desorption flux vs residence time is then recorded at a variety of temperatures between 473 and 573 K. The *p*(2 × 2) O/Pt(111) surface is prepared by dosing O_2_ from a molecular beam with a flux of 1.5 ± 0.5 ML/s for 30 min at 573 K. To ensure that O-atoms do not desorb from or diffuse out of the dosing region of the NH_3_ beam, the O_2_ dosing is continued during the transient NH_3_ desorption experiments. These conditions have been shown previously to ensure the formation of the *p*(2 × 2) O/Pt(111) structure.^20−22^ We also conducted DFT calculations of NH_3_ desorption and diffusion pathways on Pt(111) and *p*(2 × 2) O/Pt(111) using the Perdew Burke Ernzerhof (PBE) and revised Perdew Burke Ernzerhof (RPBE) functionals with and without the inclusion of D3 correction^23^ for dispersion forces—see Section 5.2.

**Figure 1:**
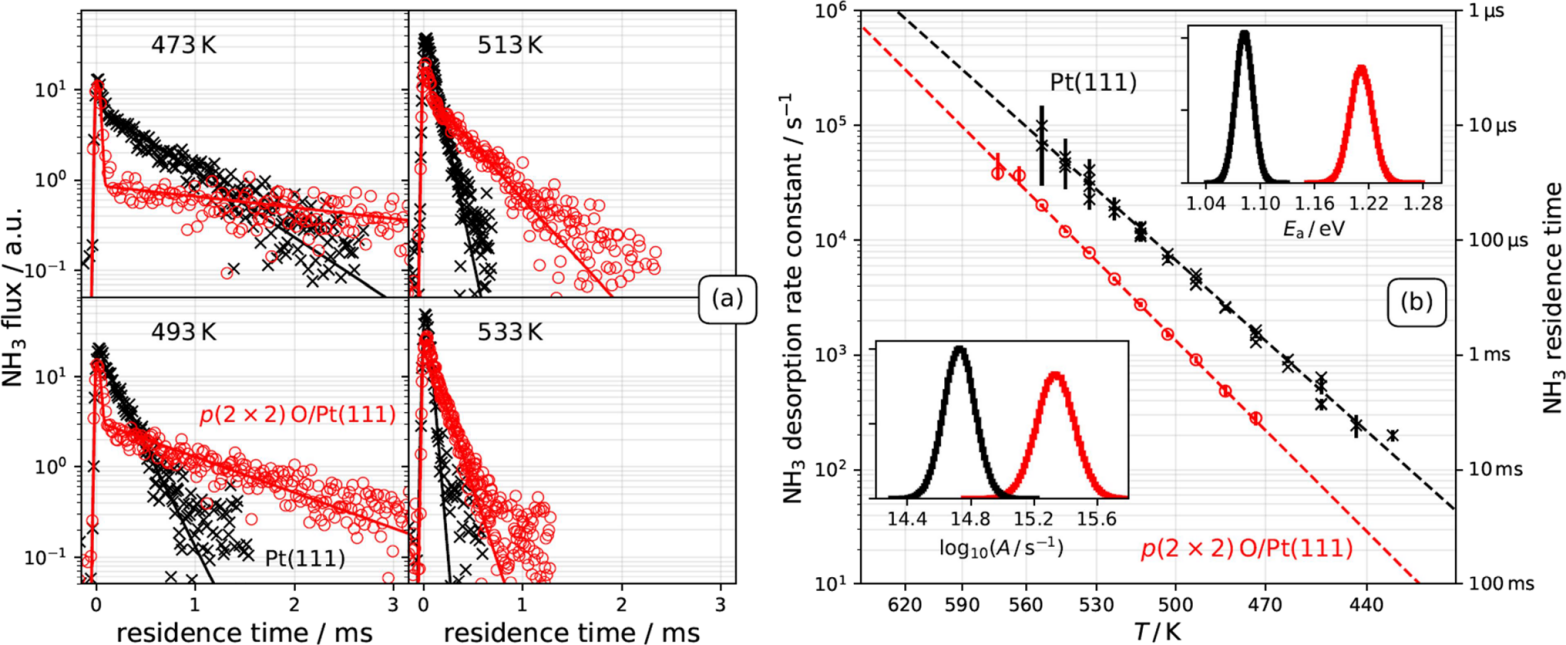
(a) Kinetic traces of NH_3_ desorbing from *p*(2 × 2) O/Pt(111) (red circles: experiment; red line: fit to eq 1) and from pristine Pt(111) (black crosses: experiment; black lines: fit to eq 1) at four surface temperatures. The temporal resolution of the experiment is ∼40 μs. All kinetic traces are obtained at constant laser power and beam flux allowing comparison of desorption yields directly—see the text. (b) Arrhenius plot of experimental NH_3_ desorption rate constants: from *p*(2 × 2) O/Pt(111) and pristine Pt(111) as obtained from data in panel (a). The dashed lines are the corresponding Arrhenius fits to the experimental rate constants. The insets show the parameter distributions (black: Pt(111); red: *p*(2 × 2) O/Pt(111)) for the activation energy *E*_a_ (upper right) and the decadic logarithm of the prefactor *A* (lower left).

Figure 1a compares typical kinetic traces of ammonia desorbing from *p*(2 × 2) O/Pt(111) and pristine Pt(111).^17^ The kinetic traces exhibit two components—temperature-independent direct scattering (DS) and temperature-dependent trapping desorption (TD). The DS component peaks at zero residence time and becomes more evident with decreasing surface temperature. This is because the TD component becomes temporally diluted due to an increased average residence time on the surface. The TD component is an exponential decay in both systems, and the VRK measurements show that NH_3_ desorption is 3–6 times slower on *p*(2 × 2) O/Pt(111), compared to the oxygen-free Pt(111). Ammonia decomposition and oxidation products (e.g., NO, N_2_, H_2_, N_2_O, and H_2_O) are absent in our experiments both for pristine and oxygen-covered Pt(111). Furthermore, we observe no variation in the ammonia desorption rate over a long exposure time, indicating that buildup of decomposition products on the surface does not take place. We conclude that the differences in NH_3_ desorption rates for the two surfaces are exclusively due to adsorbate–adsorbate interactions at Pt(111) terraces.

The kinetic traces of ammonia desorption were fit by the following expression:
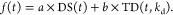
1

Here, the temperature-independent DS component follows the temporal profile of the molecular beam, with the parameter *a* describing its amplitude. The surface temperature-dependent, TD component is characterized by the amplitude *b* and desorption rate constant *k*_d_ emerging from thermally desorbing NH_3_ molecules that have been adsorbed and thermalized at the surface. We also obtain the NH_3_ desorption yield—velocity and time integrated TD contribution (∝*b*/*k*_d_ from eq 1). We find that between 473 and 573 K the desorption yield from the oxygen-covered surface amounts to 93 ± 15% of the pristine Pt(111), indicating that NH_3_’s sticking coefficient is almost unaffected by the presence of the *p*(2 × 2) O-atom overlayer. This conclusion is further supported by the indistinguishable, subthermal speed distributions of desorbing NH_3_ molecules from *p*(2 × 2) O/Pt and clean Pt(111). From the principle of detailed balance, this indicates that the initial sticking probability has the same translational energy dependence in both cases—see ref (17)—and is not affected by adsorbed O-atoms. Interestingly, these results are consistent with the observations of King and co-workers for NH_3_ sticking at O-covered Pt(100).^24^

By fitting the Arrhenius expression to the derived desorption rate constants (Figure 1b), we find that O-atom adsorption to the Pt(111) surface alters the prefactor and the activation energy, *A* and *E*_a_, for desorption from clean Pt(111)—*E*_a_ = 1.08 ± 0.02 eV and *A* = 10^14.8 ± 0.2^ s^–1^ —compared to oxygen-covered Pt(111)—*E*_a_ = 1.21 ± 0.02 eV and *A* = 10^15.3 ± 0.2^ s^–1^. The higher activation energy of NH_3_ desorption from *p*(2 × 2) O/Pt(111) indicates that it binds more strongly to the surface due to the presence of co-adsorbed O-atoms. The higher prefactor indicates that the O-atom overlayer reduces the entropy of the NH_3_ adsorbate. It is important to realize that activation energies for desorption are equal to adsorption enthalpies, which are strictly temperature-dependent quantities. By modeling the desorption prefactor, which provides the adsorbate entropy, it becomes straightforward to convert the adsorption enthalpy to a binding energy, which can be directly used to evaluate the accuracy of electronic structure methods. Surprisingly, DFT calculations employing PBE and RPBE functionals were not able to reproduce the O-induced difference in activation energies observed in the experiment. This motivated us to implement a semiempirical co-adsorbate interaction potential from which the stabilization energy and the adsorbate entropy of NH_3_ could be determined.

The main idea behind our approach to the co-adsorbate interaction potential is to describe the change in the NH_3_–Pt(111) energy landscape induced by the *p*(2 × 2) O-atom overlayer assuming that oxygen coverage does not alter the covalent NH_3_–Pt bond from what it is on clean Pt(111). This means that the changes to the NH_3_ binding energy at the surface emerge fully from attraction and repulsion by O-atoms. The covalent NH_3_–Pt(111) energies have been determined with DFT calculations of diffusion pathways using the PBE functional. We justify the use of PBE, over any other functional, because it provided almost exact agreement with experimentally derived diffusion barriers,^17^ which are crucial for a good description of the adsorbate entropy. To avoid errors introduced by the DFT-PBE-derived NH_3_/Pt(111) binding energy, we use instead the experimentally derived value^17^ in the following rate constant modeling. We assumed that noncovalent interactions between NH_3_ and O can be treated additively. The interaction of NH_3_ with adsorbed O-atoms is described by a semiempirical pair-potential which includes electrostatic, dispersive, and repulsive contributions. We refer to this noncovalent energy contribution as the NC-AIM.

**Figure 2:**
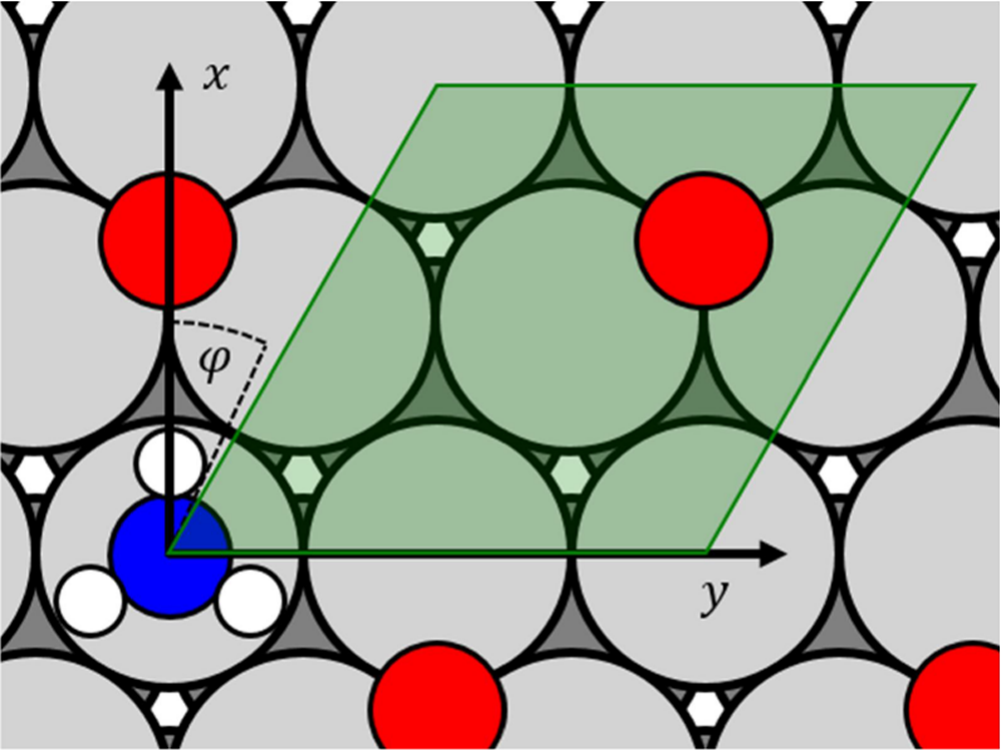
Structural model employed to characterize NH_3_ interaction energies at O-atom-covered Pt(111) using DFT calculations and the NC-AIM. The light green shaded region is the elementary cell of the *p*(2 × 2) O-atom overlayer in which the NH_3_* configurations are sampled to determine the adsorbate partition function.

The NC-AIM is explained in detail in Supporting Information (SI) Section S1; here, we provide only a brief description. In Figure 2, the underlying adsorbate structures and key coordinates of the NC-AIM are shown. In the NC-AIM, the electrostatic contributions to the NH_3_–O interactions are described by Coulomb interactions between point charges, placed at the positions of each atom of the adsorbates as well as their induced image charge positions within the metal—see also Figure S1. The effective atomic charges are parametrized using the dipole moment of gas-phase NH_3_ together with work function measurements of NH_3_^4^ and O^25^ covered Pt(111). The dispersion forces between two atoms are estimated from London theory^26^ using atomic ionization potentials and polarizability volumes of gas-phase species. To account for Pauli repulsion between two atoms at short distances, we included the *r*^–12^ term, as used in the Lennard-Jones potential. The parameters of the repulsion term were estimated using van der Waals radii of gas-phase atoms.^27^

The NC-AIM model describes the observed decrease of NH_3_ adsorbate entropy on oxygen-covered Pt(111) as a result of rotational and translational hindrance^13^ of the molecule due to interactions with O-atoms. Note that this is consistent with electron-stimulated H^+^ ion angular distributions (H^+^-ESIAD), data of Netzer and Madey that provide direct evidence for NH_3_ rotational hindrance on O-covered Ni(111).^28^ Therefore, for accurate treatment of co-adsorbate interactions in the NC-AIM, we accounted for NH_3_ in-plane displacement (*x*, *y*) and its rotation around the *C*_3_-axis (φ)—see Figure 2.

Based on the comprehensive diffusional potential energy landscape for NH_3_ on Pt(111) constructed from DFT, we used the NC-AIM to formulate the thermal desorption rate constant of NH_3_ from *p*(2 × 2) O/Pt(111).^29^ Here, the desorption rate constant is formulated as a product of the adsorption rate constant and the equilibrium constant between adsorbed and gas phases—see also ref (30). For NH_3_ desorption from *p*(2 × 2) O/Pt(111), it is given by
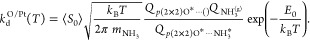
2

Here, ⟨*S*_0_⟩ and *E*_0_ are the NH_3_ thermal sticking coefficient and the binding energy at the surface, respectively. Asterisks denote adsorbates. The quotient of partition functions *Q* is crucial for accurate description of the prefactor and includes contributions from the gas-phase molecule (*Q*_NH_3_^(g)^_), the *p*(2 × 2) O-atom overlayer in the absence of ammonia (*Q*_*p*(2 × 2)O*···()_), and the *p*(2 × 2) O-atom overlayer with adsorbed ammonia (*Q*_*p*(2 × 2)O*···NH_3_^*^_).

In general, modeling desorption rate constants has in the past ignored the adsorbate-induced changes of the surface phonon spectrum.^13,31^ We also employ this assumption to model our experiment, as the influence of NH_3_ adsorption, the phonon spectrum of Pt(111) is expected to be small, due to the large mass difference between Pt and NH_3_ which results in good separability between NH_3_’s degrees of freedom (which we take into account) and Pt vibrations (which cancel out in eq 2). Furthermore, phonons are collective properties, which will remain unaffected by the low coverages of NH_3_ (≤5 × 10^–4^ ML) present in our experiment. Note that changes of the density of states associated with the O-atom overlayer are taken into account—O-atoms in close coordination to NH_3_ exhibit altered vibrational frequencies^32^—see the SI, Section S2 for details.

The analysis is simplified by modeling of the ratio of NH_3_ thermal desorption rate constants from *p*(2 × 2) O/Pt(111) and pristine Pt(111). The simplification arises as some factors appearing in eq 2, for example, sticking coefficients, cancel out when the ratio is taken. The ratio of thermal rate constants is given by eq 3.
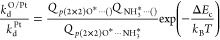
3Here, Δ*E*_c_ is the complexation energy which represents the energetic stabilization of NH_3_ induced by co-adsorbed O atoms. Of course, this rate constant ratio requires knowledge of the partition function for NH_3_* on clean Pt(111), denoted as *Q*_NH_3_^*^···()_. When computing the partition functions, all O* degrees of freedom are approximated as harmonic oscillators, which is justified by the restricted O* diffusion inside the *p*(2 × 2) structure. The required harmonic frequencies for NH_3_*, O*, and the (NH_3_–O)* complex on Pt(111) are adopted from the DFT calculations of Offermans et al.^32^ with scaling corrections employed to better reproduce experimentally available vibrational spectra of NH_3_* and O* on Pt(111)^33−35^—see the SI, Section S2 for details. The NH_3_* translational partition function on *p*(2 × 2) O/Pt(111) is determined using NH_3_–Pt(111) interaction energies from DFT calculations^17^ and the NC-AIM with the *C_3_*-axis rotation coupled to the in-plane displacement coordinates—see SI Section S2.

**Figure 3:**
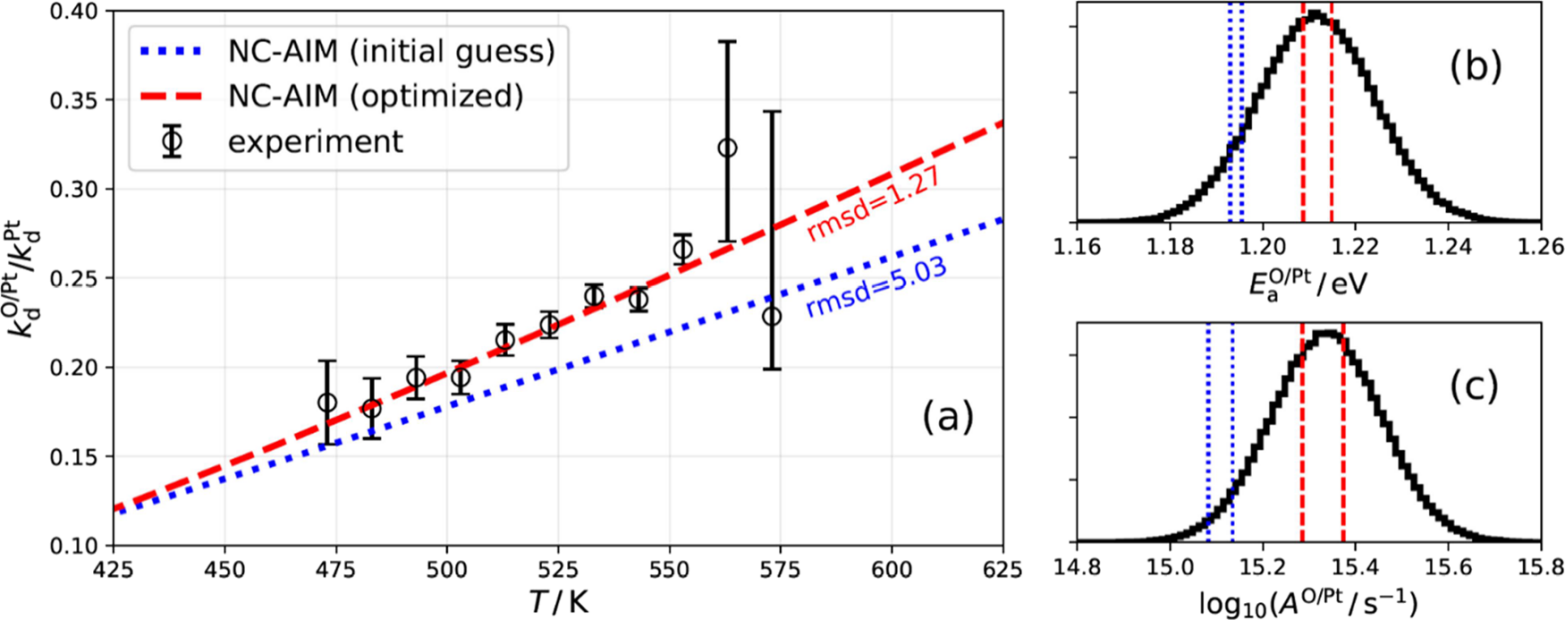
(a) Rate constant ratio from VRK experiments compared with modeling results of the NC-AIM with two sets of parameters. The root mean square deviation (rmsd) is shown for each model. The blue dotted line is the prediction of the NC-AIM based on estimated interaction parameters. Optimizing the parameters for the best fit to the experiment produces the red dashed line. Panels (b,c) show Arrhenius parameters for thermal desorption rate constants for NH_3_ desorption from O/Pt. Accurate *k*_d_^O/Pt^ values were obtained from the optimized NC-AIM rate constant ratio (eq 3) multiplied by previously determined and highly accurate values of *k*_d_^Pt^.^17^ (b) Experimentally derived Arrhenius activation energy distribution (black) compared to the NC-AIM predictions. (c) Analogous plot to (b) for the decadic logarithm of the Arrhenius prefactor. Note that the NC-AIM rate constants do not perfectly obey an Arrhenius law; hence, two vertical lines are indicated for each parametrization, to indicate the deviation from Arrhenius behavior over this temperature range.

The rate constant ratio model (eq 3) is compared to experimental results in Figure 3a. Even when using the NC-AIM prediction based on crudely estimated parameters (blue dotted line in Figure 3a), the agreement with the experiment is good. Since the estimation of dispersive and repulsive interactions was highly simplified, we optimized the associated interaction parameters to better fit the experimental rate constant ratios (red dashed line in Figure 3a). Here, we scaled the dispersion coefficient and the repulsion coefficient of the H–O and N–O interactions (see Section S1.2 in the SI); the parameters related to the electrostatic contributions to the interactions were held constant, as they rely on previous experiments that determined the adsorbate influence on the surface work function.^25,36^ The optimized NC-AIM parameters yield excellent agreement with the rate constant ratios and reproduce the temperature dependence of the measured desorption rates—see Figure 3b,c.

## 3 Discussion

The excellent agreement with the experiment clearly shows that the optimized NC-AIM yields both an accurate adsorbate entropy and binding energy for NH_3_ on *p*(2 × 2) O/Pt(111). Looking deeper into the implications of this success, we find that the NC-AIM yields a complexation energy Δ*E*_c_ of 147 meV and a rotational barrier of 84 meV. That is, the eclipsed configuration of NH_3_*, where the H atoms of the molecule are oriented toward the three nearest O* neighbors, is stabilized by 84 meV in comparison to the staggered configuration. Both the complexation energy and the rotational barrier are dominated by the dispersion forces in the NC-AIM; when dispersion forces are neglected, both Δ*E*_c_ and the rotational barrier are reduced in magnitude by ∼80%. The importance of dispersion forces suggested by the NC-AIM motivated us to carry out DFT calculations to describe the NH_3_–O interactions in this system, with and without dispersion corrections. To obtain results comparable to the low coverages found in our experiments, we used a (4 × 4) unit cell. The results of our calculations are presented in Table 1.

**Table 1:** Summary of the Energetic Parameters and Recommended Temperature-Dependent Rate Constants for Desorption and Hopping from/on Pt(111) and *p*(2 × 2) O/Pt(111) Derived in This Work^a^

energies/eV		experiment	PBE^c^	PBE-D3^c^	RPBE^c^	RPBE-D3^c^
binding energy	*E*_0_^Pt(111)^	1.13 ± 0.02^17^	0.973^17^	1.641	0.637	1.162
diffusion barrier	*W*_*x*_^Pt(111)^	0.71 ± 0.04^17^	0.70^17^			1.110
binding energy	*E*_0_^*p*(2 × 2) O/Pt(111)^	1.28_–0.02_^+0.03^	0.995	1.376	0.680	1.308
complexation energy^b^	Δ*E*_c_	0.147_–0.014_^+0.023^	0.022	–0.265	0.043	0.146
diffusion barrier	*W*_*x*_^*p*(2 × 2) O/Pt(111)^	1.10_–0.13_^+0.22^	0.947			0.969
hindered rotation barrier	*W*_φ_^*p*(2 × 2) O/Pt(111)^	0.084_–0.022_^+0.049^	0.045			0.058

recommended rate constants^d^		*Ã*/s^–1^	*n*	*Ẽ*/eV
desorption^17^	*k*_d_^Pt(111)^	5.492 × 10^16^	2.852	1.217
hopping^17^	*k*_h_^Pt(111)^	1.191 × 10^14^	0.872	0.750
diffusion^e^	*D*^Pt(111)^	0.137 cm^2^	0.872	0.750
desorption	*k*_d_^*p*(2 × 2) O/Pt(111)^	2.766 × 10^17^	3.157	1.351
hopping^f^	*k*_h_^*p*(2 × 2) O/Pt(111)^	4.979 × 10^14^	–0.476	1.146
diffusion^e^	*D*^*p*(2 × 2) O/Pt(111)^	0.764 cm^2^	–0.476	1.146

a Details on the uncertainty estimation can be found in Section S1.3 of the SI. The rate and diffusion constants are reported as parameters of the extended Arrhenius equation: *k*(*T*) = *Ã*×(298 K/*T*)^*n*^ exp(– *Ẽ*/*k*_B_*T*). Note that the temperature-dependent Arrhenius prefactor is *A*(*T*) = *Ã*×(298 K/*T*)^*n*^.

b *E*_0_^*p*(2 × 2) O/Pt(111)^ – *E*_0_^Pt(111)^.

c Calculated energies do not include zero-point energy correction.

d Deviation due to approximated form is ≤1% between 298 and 1300 K.

e Details on the conversion of the hopping to diffusion coefficient are provided in the SI, Section S3.1.

f Estimated from the transition-state theory using the NC-AIM barrier and RPBE-D3 frequencies—see the SI, Section S3.2.

DFT-GGA results that neglect dispersion interactions, i.e., PBE and RPBE functionals, give complexation energies Δ*E*_c_ of only 22 and 43 meV, respectively; this is inconsistent with the experiment, Δ*E*_c_ = 147_–14_^+23^ meV. These values are however remarkably close to the electrostatic contribution to the NH_3_ stabilization obtained from the NC-AIM—22 meV. Analogous DFT-GGA calculations using the D3 van der Waals correction^23^ to PBE and RPBE functionals gave Δ*E*_c_= −265 and 146 meV, respectively. Results of PBE-D3 are even in qualitative disagreement with the experiment. This finding is consistent with previous work, which found that PBE-D3 has poor performance when it comes to the description of noncovalent interactions at metal surfaces.^37^ RPBE-D3 results compare well with the experiment, supporting the conclusions from the NC-AIM that dispersion forces are critical to describing the NH_3_–O interactions for NH_3_ adsorbed on *p*(2 × 2) O/Pt(111).

It is also interesting to compare the performance of the chosen exchange–correlation functionals for the description of similar systems. Ammonia on Cu(100) is a rare example for an experimental binding energy for this molecule on a transition metal surface, determined with chemical accuracy.^38^ Here, the adsorption enthalpy (0.61 ± 0.02 eV) was reported at ∼230 K, a temperature that is low enough that the binding energy is likely to be within ∼0.02 eV of this value. Calculations with PBE and RPBE both under-bind NH_3_ to Cu by 0.18 and 0.40 eV, respectively.^39^ The under-binding errors are quite similar for NH_3_/Pt(111)—0.16 eV for PBE and 0.51 eV for RBPE shown in Table 1. These comparisons suggest further that the VRK-derived binding energies for NH_3_ on Pt are within chemical accuracy. Finally, we point out that RPBE-D3 calculations for NH_3_/Cu(100) gave a binding energy within 1 kcal/mol of the experiment.^40^ Our calculations show that NH_3_ binding to both Pt(111) and *p*(2 × 2) O/Pt(111) is also predicted accurately with this functional.

**Figure 4:**
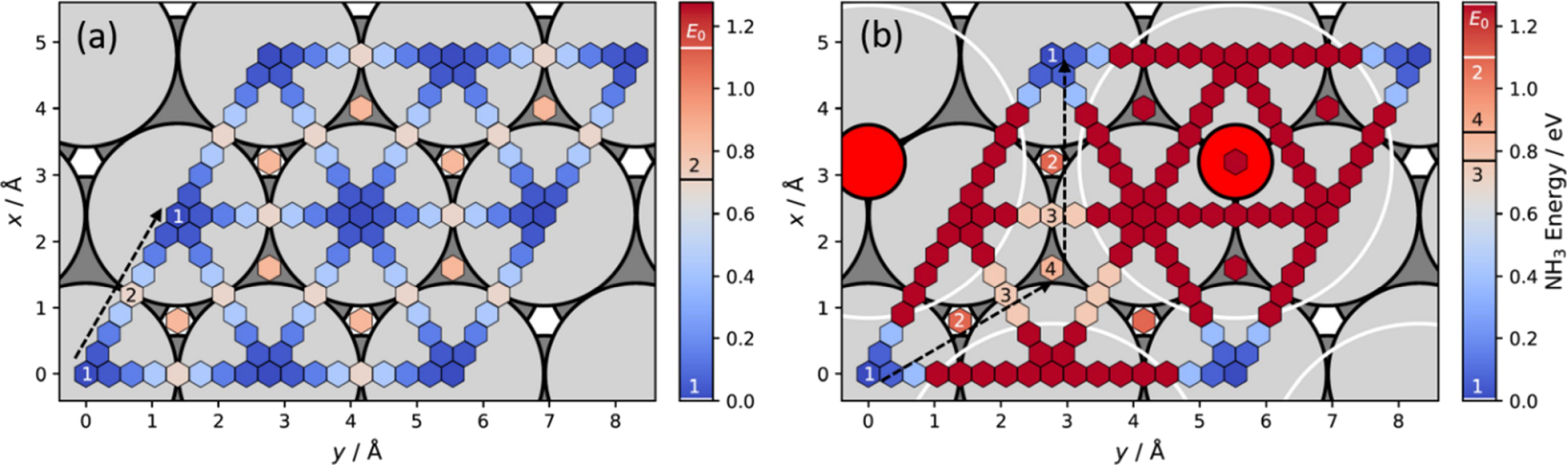
(a) Potential energy surface (PES) of NH_3_ on clean Pt(111) derived from DFT calculations from ref (17). The preferred diffusion pathway is indicated by the black dashed arrow and proceed as *top-bri-top*. The diffusion barrier is 0.71 ± 0.04 eV. (b) PES of NH_3_ on *p*(2 × 2) O/Pt(111) resulting from the sum of DFT energies (see panel (a)) and the NC-AIM with optimized parameters. The large red circles indicate the position of the oxygen atoms with white circles indicating regions around each O atom with strong O–NH_3_ repulsion. The diffusion (indicated by the black dashed arrows) follows the *top(1)-fcc(2)-bri(3)-hcp(4)-bri(3)-fcc(2)-top(1)* pathway with a barrier of 1.10_–0.13_^+0.22^ eV. The energies of relevant binding sites are indicated in the color bar. The energies in each panel are set relatively to the minimum energy of the corresponding PES, and the binding energies are indicated in the color bar as *E*_0_.

The success of the NC-AIM encouraged us get a deeper look at the diffusional potential energy surface for NH_3_ on O-covered Pt(111). In Figure 4, the energies of the NH_3_ molecule are shown for various in-plane positions on clean Pt(111)—panel (a)—and for the same positions on *p*(2 × 2) O/Pt(111)—panel (b). From the NC-AIM, we deduced a strong repulsion between N and O (see also Figure S2 in SI Section S1.2). This repulsion stems from the fact that O is coordinated to three Pt atoms, when bound at the fcc hollow sites of Pt(111);^41^ this reduces the propensity of those three Pt atoms to form Pt–N bonds with NH_3_. The white circles in Figure 4b show these N–O repulsive regions. This effectively means that a single O* blocks three out of four on-top binding sites that would have been available to NH_3_ on Pt(111). Of course, this lowers the configurational entropy of NH_3_ compared to the Pt(111) surface.

The elimination of binding sites goes hand in hand with a strong steric hindrance of site-to-site hopping pathways, previously available for NH_3_* on clean Pt(111). The same N–O repulsive regions, indicated by the white circles in Figure 4b, prevent the molecules from passing over the nearest bridge site, a pathway preferred on clean Pt(111) (black dashed arrow in Figure 4a). This forces hopping to occur through highly coordinated sites, where NH_3_* binding to the surface is substantially weakened. A hopping pathway on *p*(2 × 2) O/Pt(111) tends to involve a *top-fcc-bri-hcp-bri-fcc-top* sequence (see black dashed arrows in Figure 4b). Traveling along this path traverses a barrier of ∼1.1 eV, which is almost 0.4 eV higher than the diffusion barrier found for NH_3_ on clean Pt(111).^17^

Despite an underestimated binding energy, PBE predicts diffusion barriers in good agreement with the experiment—see Table 1. This finding is reasonable since repulsive interactions between NH_3_ and O dominate the increased diffusion barrier on *p*(2 × 2) O/Pt(111), which can be well captured even without inclusion of dispersion forces. Interestingly, RPBE-D3 yields not only the correct binding energies of NH_3_* at Pt(111) and *p*(2 × 2) O/Pt(111) but also predicts the diffusion pathway and diffusion barriers for O/Pt(111) of 0.97 eV, in good agreement with the value derived from the experiment, using the NC-AIM. However, the diffusion barrier on clean Pt(111) is substantially overestimated compared to the experiment, indicating that subtle error compensation is involved in many apparently accurate DFT results.

We close the discussion with a word of caution about the NC-AIM model. While we find that the optimized NC-AIM parameters agree quite well with the range of reported N–O and H–O interaction parameters known from the literature (see Table S1 and Section S1.3 in the SI), we do not claim to provide the best dispersion coefficients or universally applicable interaction parameters. In fact, the exact values of the interaction parameters are quite uncertain from our fit and suffer from large correlation errors between the attractive and repulsive coefficients. Nonetheless, the derived energetic parameters—binding energy, rotational barrier, and diffusion barrier—are much less affected by these uncertainties—see Table 1. This is because the rate constant ratios are much more closely related to energy differences, which profit from error cancelation between attractive and repulsive terms of the interaction potential.

## 4 Conclusions

In this work, we have reported transient desorption rates of ammonia from *p*(2 × 2) O/Pt(111) between 473 and 573 K. We found that the adsorption of O-atoms on Pt(111) reduces the NH_3_ desorption rate. The absence of NH_3_ reactions offered us the opportunity to exclusively characterize the nonreactive interaction between O* and NH_3_*. The thermal desorption rates clearly indicated an energetic stabilization of NH_3_* and a reduced adsorbate entropy on *p*(2 × 2) O/Pt(111) compared to Pt(111). We developed an NC-AIM of attractive and repulsive interactions between NH_3_* and O* which gave an accurate fit to the experimental results. The NC-AIM allowed the determination of valuable benchmark data reported in Table 1, but most importantly, the solid basis of the NC-AIM allowed us to quantify the steric hindrance of O-atoms on the diffusion of NH_3_. Compared to clean Pt(111) which already exhibits a large diffusion barrier, the NH_3_ diffusion barrier is increased further by 0.39_–0.14_^+0.22^ eV on *p*(2 × 2) O/Pt(111). Additionally, from the NC-AIM, we find that attractive interactions between NH_3_ and O-atoms are dominated by dispersion forces, a result which we could confirm with DFT calculations. Specifically, the RPBE-D3 functional appears to be the best choice for the nonreactive description of NH_3_–O interaction on Pt(111). It definitely should be considered for microkinetic modeling NH_3_ oxidation on Pt, required for the description of the Ostwald process. The findings reported here provide a solid foundation for a detailed kinetics study of NH_3_ oxidation on stepped Pt surfaces.

## 5 Methods

### 5.1 Experimental Section

The experimental apparatus has been described earlier in detail.^15,16,42^ A supersonic molecular beam of NH_3_ (∼1% NH_3_ in He, 6 bar backing pressure) was generated with a piezo-electrically driven pulsed valve at 25 Hz. The beam passed through two differentially pumped stages and entered the surface-scattering chamber, held at a base pressure of 2 × 10^–10^ mbar, impinging the Pt(111) surface (MaTeck GmbH) at an incidence angle of 30° from the surface normal. The temporal duration of the pulsed beam had a full width at half maximum of ∼40 μs. The mean kinetic energy of NH_3_ in the beam was ∼0.35 eV. The surface was prepared by sputtering with Ar^+^ (3 keV) for 10 min and subsequent annealing at 1300 K for 20 min. The cleanliness of the sample was verified with Auger electron spectroscopy. The step density of the Pt(111) crystal is estimated, based on its cut-angle accuracy, to be 0.1–0.2%. The desorbing NH_3_ is detected, 20 mm from the surface, using nonresonant multiphoton ionization accomplished with a Ti:Sapphire laser (35 fs, 0.3 W at 1 kHz). A pulsed homogeneous electric field, formed between two parallel flat meshes (repeller and extractor), projects the ions onto a pulsed MCP detector. The mass-to-charge ratio of the ions is selected by delaying the MCP pulse with respect to the pulsed field extraction. The ion image is obtained from the phosphor screen at the back of the MCP detector using a CCD camera.

Prior to the experiments, O_2_ is dosed (1.5 ± 0.5 ML/s) from a second molecular beam (500 Hz), at normal incidence, for 30 min at the Pt(111) surface at 573 K. This condition is sufficient to establish the *p*(2 × 2) structure of oxygen atoms with 0.25 ML at Pt(111). The O_2_ beam was continuously operated during desorption experiments to prevent any O-coverage dilution as a consequence of O-atom diffusion or recombinative desorption. These conditions were found earlier to ensure a stable *p*(2 × 2) structure.^20−22^ Due to the absence of NH_3_ reaction and due to the small NH_3_ coverages per molecular beam pulse (∼5 × 10^–4^ ML), we assume that the *p*(2 × 2) structure of O-atoms on Pt(111) is not perturbed during the experiments.

The kinetic traces—defined as the flux of NH_3_ departing from the surface vs residence time—have been obtained according to the procedure, documented in detail in our previous work.^15,16^ Briefly, the density ion images are corrected for the thermal background, separated from the signal by its velocity, and ion images are converted to flux. The positions of the image are transformed to velocities with the ion’s time-of-flight from the laser focus to the detector. The flux images at each beam-laser delay are integrated for velocities between 500 and 1000 m/s, close to the surface normal. By using this velocity range, we suppress DS contributions in the kinetic traces. The beam-laser delay time-axis is corrected by NH_3_’s time of flight from the surface to the laser focus in order to extract its residence time at the surface.

### 5.2 DFT Calculations

NH_3_ interactions with Pt(111) and *p*(2 × 2)O/Pt(111) surfaces have been modeled using the Vienna ab initio simulation package.^43−47^ Periodic DFT calculations were performed at the level of GGA using the PBE^48^ and the RPBE^49^ exchange–correlation functionals. In addition, the systems have been characterized using these functionals and including the D3 dispersion correction (PBE-D3 and RPBE-D3) from Grimme and coworkers.^23^

The core–electron interaction is approximated by the projector augmented wave potentials.^50,51^ A cutoff energy for a plane-wave basis of 400 eV is applied in the case of the clean Pt(111) surface, whereas for *p*(2 × 2)O/Pt(111), it is set to 765.5 eV. Both the Pt(111) and the *p*(2 × 2)O/Pt(111) surfaces are modeled by a (4 × 4) unit cell and 4-layer slab, and the two lowest Pt layers are kept fixed. A 24 Å vacuum region was added to the slab to avoid interaction between periodic images in the *z*-direction. The Brillouin zone was sampled with an 8 × 8 × 1 and 3 × 3 × 1 Γ-centered grid of special k-points for the Pt(111) and the *p*(2 × 2)O/Pt(111) surfaces, respectively.

To predict adsorption energies, the two topmost surface layers, the NH_3_ molecule, and O atoms, whenever present, were allowed to relax until forces were lower than 0.02 eV/Å. The reaction paths and transition states for diffusion and desorption were identified by the climbing image variant of the nudged elastic band method.^52^ The calculation was considered converged when forces were <0.05 eV/Å.

## Supporting Information Available

Formalism and construction of NC-AIM, parametrization of point charges, parametrization of dispersive and repulsive contributions, uncertainty estimation of NC-AIM-derived energies, details on the modeling of rate constant ratios, modeling of hopping rate constants with harmonic TST, and relationship between hopping and diffusion coefficients (PDF)
