# Supplementary material for: Steric Hindrance of NH3 Diffusion on Pt(111) by Co-Adsorbed O-Atoms
Source: J Am Chem Soc. 2022 Nov 18;144(47):21791–9. doi: 10.1021/jacs.2c10458 (PMC9716551; doi:10.1021/jacs.2c10458)
Supplement: Supplementary file 1 — ja2c10458_si_001.pdf [file ja2c10458_si_001.pdf]

Supporting Information

for

Steric hindrance of NH_3 diffusion on Pt(111) by co-adsorbed O-atoms

Dmitriy Borodin[1,2]*, Oihana Galparsoro[3,4], Igor Rahinov[5], Jan Fingerhut[1], Michael Schwarzer[1], Stefan Hörandl[1], Daniel J. Auerbach[2], Alexander Kandratsenka[2], Dirk Schwarzer[2], Theofanis N. Kitsopoulos[1,2,6,7]** and Alec M. Wodtke[1,2,8]***

[1]Institute for Physical Chemistry, Georg-August University of Goettingen, Tammannstraße 6, Goettingen 37077, Germany.

[2]Department of Dynamics at Surfaces, Max Planck Institute for Multidisciplinary Sciences, Am Fassberg 11, Goettingen 37077, Germany.

[3]Donostia International Physics Center (DIPC), Paseo Manuel de Lardizabal 4, Donostia-San Sebastián 20018, Spain.

[4]Kimika Fakultatea, Euskal Herriko Unibertsitatea UPV/EHU, P.K. 1072 Donostia-San Sebastián 20018, Spain.

[5]Department of Natural Sciences, The Open University of Israel, Raanana 4353701, Israel.

[6]Department of Chemistry, University of Crete, Heraklion 71500, Greece

[7]Institute of Electronic Structure and Laser – FORTH, Heraklion 70013, Greece

[8]International Center for Advanced Studies of Energy Conversion, Georg-August University of Goettingen, Tammannstraße 6, Goettingen 37077, Germany.

Email: *dborodi@gwdg.de, **theo.kitsopoulos@mpinat.mpg.de, ***alec.wodtke@mpinat.mpg.de

S1. Construction of the Non-Covalent Additive Interaction Model (NC-AIM)

The main idea behind the Non-Covalent Additive Interaction Model (NC-AIM) is to construct a physical description of the change in the NH_3-Pt(111) energy landscape induced by the $p(2 \times 2)$ O-atom overlayer. We assume that the interaction energy of NH_3 on $p(2 \times 2)$ $\mathrm{O/Pt(111)}$ ($V_{\mathrm{NH3-O/Pt}}$) is well approximated by the sum of NC-AIM energy ($V_{\mathrm{NH3-O}}^{\mathrm{NC-AIM}}$) and NH_3-Pt(111) potential energy surface ($V_{\mathrm{NH3-Pt}}^{\mathrm{DFT}}$) characterized with DFT in our previous work[1]:

$$V_{\mathrm{NH3-O/Pt}} \approx V_{\mathrm{NH3-O}}^{\mathrm{NC-AIM}} + V_{\mathrm{NH3-Pt}}^{\mathrm{DFT}}. \qquad \text{(S1)}$$

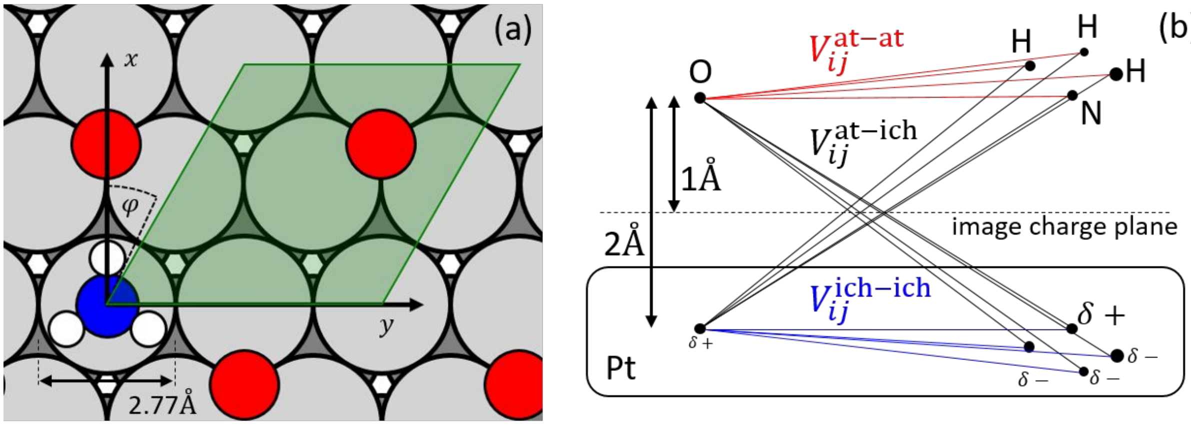

Figure S1: **(a)** The structural model employed to characterize NH_3 interaction energies at O-atom covered Pt(111) using DFT calculations and the non-covalent additive interaction model (NC-AIM). The light green shaded region is the elementary cell of the $p(2 \times 2)$ O-atom overlayer in which the NH_3^* configurations are sampled to determine the adsorbate partition function. **(b)** Distribution of point charges in the NC-AIM along with key geometries of the adsorbates. N- and O-atoms are located at fixed z-distance of 1 Å from image charge plane. This distance was estimated based on half of the typical atom-metal bond length. The adsorbed ammonia molecule is assumed to retain its geometry from the gas phase. The lines indicate relevant pair-interactions contributing to NC-AIM.

Figure S1 (a) shows the underlying adsorbate structures and key coordinates of NC-AIM. It is well known that an accurate description of translational and rotational degrees of freedom is required for reliable prediction of adsorbate entropies[2]. The presence of the O-atom overlayer has the largest influence on the molecule's in-plane displacement and its rotation about the C_3-axis. This can be understood considering that potential NH_3 binding sites become blocked by O-atoms and previously free C_3-axis rotation becomes hindered by attractive interactions between H and O atoms.

We assume that adsorbed ammonia molecule NH_3^* retains its gas-phase structure and make use of a static (2×2) $\mathrm{O/Pt(111)}$ surface approximation. The non-covalent interactions between NH_3 and O are described by a semi-empirical pair-potential which includes an electrostatic contribution, V_{elst}, dispersion forces, V_{disp}, and Pauli repulsion terms V_{rep}. The pair-potential between atoms i and j is given by:

$$V_{ij}^{\mathrm{at-at}} = V_{\mathrm{elst}}\left(\left|\overrightarrow{r_{ij}}\right|\right) + V_{\mathrm{disp}}\left(\left|\overrightarrow{r_{ij}}\right|\right) + V_{\mathrm{rep}}\left(\left|\overrightarrow{r_{ij}}\right|\right). \qquad \text{(S2)}$$

We also include the interactions between an atom (at) and an image charge (ich), induced by another atom, as well as the interactions between two image charges. This is included within the electrostatic potential only:

$$V_{ij}^{\mathrm{at-ich}} = V_{\mathrm{elst}}\left(\left|\overrightarrow{r_{ij}}\right|\right), \tag{S3}$$

$$V_{ij}^{\mathrm{ich-ich}} = V_{\mathrm{elst}}\left(\left|\overrightarrow{r_{ij}}\right|\right). \tag{S4}$$

In Fig. S1 (b) we show all interaction terms considered to describe the non-covalent interaction energy between an adsorbed NH_3 molecules and a single adsorbed O-atom.

In total 16 terms have to be summed up, where 4 describe the atom-atom interaction, 8 consider atomic charge and image charge interaction and 4 are for the image charge-image charge interactions. The total energy of NC-AIM is given by:

$$V_{\mathrm{NH3-O}}^{\mathrm{NC-AIM}} = \sum_{i,j \neq i} V_{ij}^{\mathrm{at-at}} + \sum_{i,j \neq i} V_{ij}^{\mathrm{at-ich}} + \sum_{i,j \neq i} V_{ij}^{\mathrm{ich-ich}}. \tag{S5}$$

The interaction of atomic charges with their own image charges is not included as these are part of the covalent interaction determined from the DFT PES. The parametrization and implementation of individual contributions in Eq. S2 is described in Sections S1.1 and S1.2.

S1.1. Parametrization of electrostatic interactions

In NC-AIM the electrostatic contributions are described by Coulomb interaction between two point charges, given by:

$$V_{\mathrm{elst}}^{\mathrm{C}}\left(\left|\overrightarrow{r_{ij}}\right|\right) = \frac{1}{4\pi\varepsilon_0}\frac{q_i\, q_j}{\left|\overrightarrow{r_i} - \overrightarrow{r_j}\right|}, \tag{S6}$$

with q and $\vec{r}$ being the charge and position vector of the point charge, respectively. These point charges are placed at the positions of the atoms of the adsorbates—H, N and O—and also on the induced image charges within the bulk metal—see Fig. S1(b). The N and O atoms are placed both at a distance of 1Å from the image charge plane. The partial charges of the atoms are parametrized in two steps. In the first step, the partial charges inside the NH_3 molecule are distributed based on the dipole moment of the gas phase molecule (1.47 D)[3], resulting in $q_N = -0.945\ e$ and $q_H = -q_N/3$, with e being the elementary charge. No charge is assigned to the O-atom in the first step. The second step makes a charge assignment to the adsorbate based on the experimentally observed work function changes of the Pt(111) surface upon adsorption of O[4] and NH_3[5]. Both adsorbates, NH_3 and O, have a saturation coverage of 0.25 ML and lead to a work function change of -2.8 [5] and $+0.3$ eV [4] at these conditions, respectively. When molecules adsorb at a metal they tend to draw or push charge into the metal, which leads to a change of the work function as a result of an induced dipole moment μ_s at the metal interface. This relation is described by:

$$\Delta\Phi_s = n_s \frac{\mu_s}{\varepsilon_0}. \tag{S7}$$

Here, n_s is the atom/molecule density to which the work function change $\Delta\Phi_s$ refers to. Using Eq. S7 the O atom receives a negative partial charge of $-0.012\ e$. The positive partial charge of NH_3 is distributed between the atoms based on the Pauling electronegativity value, i.e. smaller electronegativity leads to higher positive charge. The partial charges determined this way are 0.350 and -0.919 e for H and N, respectively. To describe the electrostatic interactions of NH_3* with O* we have considered interactions for distances up to 350 Å where all interactions are converged below 0.5 meV.

S1.2. Implementation of dispersive and repulsive contribution

In addition to the electrostatic interactions, we have also included dispersion forces which are known to contribute to the binding energy of many molecules on surfaces and to hydrogen bonded systems in the gas phase[2, 6-7]. We treat dispersion as simplified pair-interactions between two atoms using the London formula[8] given by:

$$V_{\text{disp}}^{\text{L}}\left(\left|\overrightarrow{r_{ij}}\right|\right) = -\underbrace{\frac{3}{2}\frac{IP_i\,IP_j}{IP_i + IP_j}\alpha_i\,\alpha_j}_{C_6^L}\frac{1}{\left|\overrightarrow{r_i} - \overrightarrow{r_j}\right|^6}. \quad \text{(S8)}$$

Ionization potentials values IP, for gas-phase H, N and O were used[3]. The polarizability volume α for an O-atom is also taken from gas phase measurements—0.8 Å^3 [3]. The polarizability volumes assigned to N and H inside the molecule are 0.75 and 0.45 Å^3, which sum up to the polarizability volume of gas phase NH_3, 2.1 Å^3. The total molecule's polarizability was distributed based on the ratios of gas phase atom polarizabilities[3]. The dispersion interactions converged including interactions out to a distance of 10 Å.

To account for repulsive interactions between two atoms at short distances we included the r^{-12} term, as used in the Lennard-Jones potential:

$$\frac{V_{\text{rep}}^{\text{LJ}}\left(\left|\overrightarrow{r_{ij}}\right|\right)}{\text{eV}} = \left(\frac{d_{12}}{\left|\overrightarrow{r_i} - \overrightarrow{r_j}\right|}\right)^{12}. \quad \text{(S9)}$$

Unfortunately, to the best of our knowledge, there is no generally accepted scheme for choosing the repulsion parameter d_{12}. It is known that typical bond distances between two atoms are 50-70% of the sum of their van der Waals radii. As an initial guess for H-O interactions, we choose d_{12} such that $V_{ij}^{\text{at}-\text{at}} = 0$ at 60% of the sum of H and O-atom vdW radii. The same procedure was used for N-O interaction, but we choose d_{12} such that $V_{ij}^{\text{at}-\text{at}} = 0.045$ eV at 60% of the sum of N and O-atom vdW radii to account for the strong repulsive character of the N-O interaction due to their repulsive electrostatic contribution. In Fig. S2 we show the initially determined alongside optimized pair-potentials for H-O and N-O.

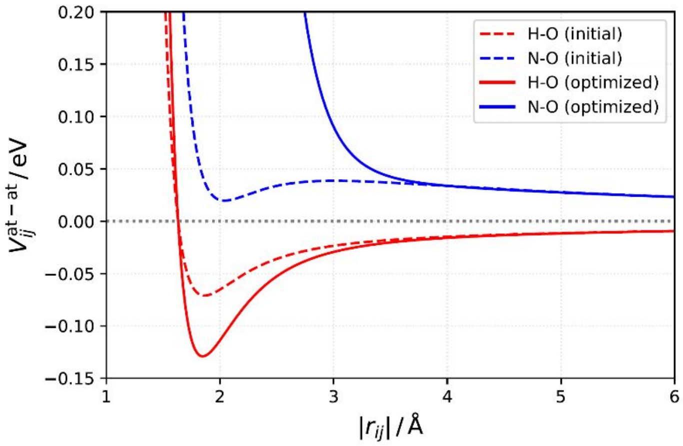

Figure S2: H-O and N-O pair potentials predicted from methods described in the text (dashed lines) and optimized to the experimental desorption rate constant ratio (solid lines).

We found that the initial parametrization of NC-AIM did not reproduce the magnitude and temperature dependence of the rate constant ratio perfectly. However, the decent agreement, given the simplicity of parameter estimation, implies the model possesses realistic physical basis. To ensure more accurate reproduction of the experimental rate constant ratios, we optimized the dispersion and repulsion parameters only. This ensures that the model still predicts the proper change of work function induced by NH_3 or O atom adsorption[4-5]. Specifically, we optimized the C_6^L coefficient for H-O and N-O dispersion using a single scaling factor. Simultaneously, we freely optimized the d_{12} coefficient for N-O repulsion, while the d_{12} coefficient of H-O was optimized to maintain the condition $V_{ij}^{\mathrm{at-at}}(0.6 \times (r_{\mathrm{vdW}}^{\mathrm{H}} + r_{\mathrm{vdW}}^{\mathrm{O}})) = 0$. The pair potentials resulting from the optimization procedure are also shown in Fig. S2. All initial and optimized parameters of the NC-AIM are listed in Table S1.

Table S1: Dispersion and repulsion parameters used for NC-AIM. Optimized parameters provide best reproduction of experimental observations. The electrostatic interactions are described by partial point charges on H, N and O which are $0.350\ e$, $-0.919\ e$ and $-0.012\ e$, respectively. For comparison literature-reported interaction parameters ranges[9-10] are provided—see Sec. S1.3 for details.

Parameter ↓ \ Pair-interaction →	H - O	N - O
C_6^L/Å^{6}eV	3.672	6.316
d_{12}/Å	1.443	1.554
C_6^{opt}/Å^{6}eV	$8.08^{+4.77}_{-2.20}$	$13.90^{+8.21}_{-3.79}$
d_{12}^{opt}/Å	$1.530^{+0.114}_{-0.072}$	$2.380^{+0.161}_{-0.142}$
C_6^{Lit}/Å^{6}eV	4.93 – 8.59	15.86 – 24.67
d_{12}^{Lit}/Å	1.79 – 1.97	2.14 – 2.30

S1.3. Uncertainty estimation of NC-AIM derived energies

We optimized the dispersion (C_6) and repulsion (d_{12}) parameter of H-O and N-O pair interactions in order to reproduce the rate constant ratio shown in Fig. 3 of the main text. The uncertainties of the derived energetic parameters—complexation energy, rotational barrier and diffusion barrier arise from the correlated uncertainty of the fitted dispersion and repulsion parameters—see Table S1. We investigated how much the potential parameters can be changed such that 1) the magnitude of the rate constant ratio remains reproduced and 2) the NC-AIM predicted Arrhenius prefactor and activation energy for desorption from $p(2 \times 2)\ \mathrm{O/Pt(111)}$ still remain within the $\pm 1\sigma$ region of the derived parameter distribution (see Fig. 3 insets). Potential parameters reproducing $+1\sigma$ and -1σ region of the 1D Arrhenius parameter distributions are used to re-evaluate ΔE_c, $W_x^{p(2\times 2)\ \mathrm{O/Pt(111)}}$, and $W_\varphi^{p(2\times 2)\ \mathrm{O/Pt(111)}}$. Their deviation from the best fit parameters (red dashed line of Fig 3) is defined as the uncertainty of the experimentally derived energies and is given in Table 1 of the main text.

We have also inspected the literature for typical H-O and N-O interaction parameters, which are summarized in Table S1. Typically values for the ε and σ parameters of a Lennard-Jones potential were reported[9-10], which can be easily converted to the C_6 and d_{12} coefficients of the NC-AIM via $C_6 = 4\varepsilon\sigma^6$ and $d_{12} = \sqrt[12]{4\varepsilon\sigma^{12}}$. We find that our interaction parameters derived from the NC-AIM optimization, are in reasonable agreement with previously reported parameter ranges, indicating that our analysis is physically sound.

S2. Details on the modelling of $k_d^{\mathrm{O/Pt}}/k_d^{\mathrm{Pt}}$

The modelling of the thermal rate constant ratio relies on the idea to express an equilibrium constant between adsorption and desorption state. The product of this equilibrium constant with the adsorption rate constant yields the desorption rate constant (Eq. 2 of the main text). See Ref. 11 for further details. We aim to model the desorption rate constant ratio of NH_3 from $p(2\times 2)$ $\mathrm{O/Pt}$ (111) and Pt(111) given in Eq. 3 of the main text, see also below:

$$\frac{k_d^{\mathrm{O/Pt}}}{k_d^{\mathrm{Pt}}}=\frac{Q_{p(2\times 2)\mathrm{O}^*\cdots()}Q_{\mathrm{NH}_3^*\cdots()}}{Q_{p(2\times 2)\mathrm{O}^*\cdots\mathrm{NH}_3^*}}\exp\left(-\frac{\Delta E_{\mathrm{c}}}{k_{\mathrm{B}}T}\right). \quad (3)$$

As mentioned in the main text we find no evidence that the sticking probability of NH_3 has changed due to adsorption of O-atoms. Thus, it cancels out in Eq. 3.

A crucial part of our analysis is to use appropriate approximations to describe the partition functions. The formulation of the model as a rate constant ratio helps to make this approach more accurate, as the ratio profits from error cancelation and many of the partition function ratios are 1. Although surface contributions to the partition functions are conventionally excluded in the modelling of desorption rates, it cannot be fully excluded in this particular case. In the work of Offermans *et al.*[12] it was shown that three O-atoms in closest coordination to the NH_3 exhibit frequencies deviating from O-atoms not complexed to ammonia. This must be explicitly accounted for in our problem.

The partition function $Q_{p(2\times 2)\mathrm{O}^*\cdots\mathrm{NH}_3^*}$ includes contribution from the $p(2\times 2)$ O-atom overlayer and NH_3* adsorbed within the O atom lattice, while the partition function $Q_{p(2\times 2)\mathrm{O}^*\cdots()}$ describes the $p(2\times 2)$ O-atom overlayer in the absence of NH_3 on the surface. The fraction of both partition functions can be simplified to contributions of only three neighboring O-atoms inside $p(2\times 2)$ and of NH_3 surrounded by three O-atoms inside $p(2\times 2)$. We can further assume that the partition function of NH_3 and the O-atoms is separable. This leads to Eq. S10:

$$\frac{k_{\mathrm{d}}^{\mathrm{O/Pt}}}{k_{\mathrm{d}}^{\mathrm{Pt}}}=\frac{Q_{\mathrm{O}^*\cdots()}^3\;\;Q_{\mathrm{NH}_3^*\cdots()}}{Q_{\mathrm{O}^*\cdots(\mathrm{NH}_3^*)}^3Q_{\mathrm{NH}_3^*\cdots(\mathrm{O}^*)}}\exp\left(-\frac{\Delta E_{\mathrm{c}}}{k_{\mathrm{B}}T}\right). \quad (\mathrm{S10})$$

The indices A ⋯ (B) indicate the partition function of A with B as neighbor. Due to restricted mobility of O atoms inside the $p(2\times 2)$ overlayer, the harmonic approximation is suitable for the description of O-atom DOFs. The ratio of the O-atom partition functions is then given by:

$$\frac{Q_{\mathrm{O}^*\cdots()}^3}{Q_{\mathrm{O}^*\cdots(\mathrm{NH}_3^*)}^3}=\frac{\left(q_x^{\mathrm{O}^*\cdots()}q_y^{\mathrm{O}^*\cdots()}q_z^{\mathrm{O}^*\cdots()}\right)^3}{\left(q_x^{\mathrm{O}^*\cdots(\mathrm{NH}_3^*)}q_y^{\mathrm{O}^*\cdots(\mathrm{NH}_3^*)}q_z^{\mathrm{O}^*\cdots(\mathrm{NH}_3^*)}\right)^3}. \quad (\mathrm{S11})$$

Each degree of freedom (x, y and z) of the three O-atoms surrounding the NH_3 molecule are described by the harmonic oscillator partition function:

$$q_i(T) = \frac{1}{1 - \exp\left(-\frac{h\nu_i}{k_B T}\right)}. \qquad \text{(S12)}$$

The ratio of $Q_{\mathrm{NH}_3^* \cdots ()}$ and $Q_{\mathrm{NH}_3^* \cdots (\mathrm{O}^*)}$ requires in principle the inclusion of 12 degrees of freedom; however, previous work has demonstrated that the six internal harmonic frequencies of NH_3 change negligibly upon complexation with O-atoms[12]. Consequently, the ratio of harmonic partition functions describing the internal vibrational modes yield negligible (<<1%) changes to the rate constant ratio up to 1200 K and are therefore ignored. The remaining six degrees of freedom are associated with rotational and translational motion. The hindered rotation of NH_3 around the in-plane coordinates—R_x and R_y—is well described by harmonic oscillators given the high vibrational frequency ($> 600\ \mathrm{cm}^{-1}$) and likely a high rotational barrier along this axis[13-14]. Also the hindered translational motion along the surface normal (z) is approximated as harmonic oscillator. The ratio of the NH_3 partition functions is given by:

$$\frac{Q_{\mathrm{NH}_3^* \cdots ()}}{Q_{\mathrm{NH}_3^* \cdots (\mathrm{O}^*)}} = \frac{q_z^{NH_3^* \cdots ()} q_{R_x}^{NH_3^* \cdots ()} q_{R_y}^{NH_3^* \cdots ()} \quad q_{xy\varphi}^{NH_3^* \cdots ()}}{q_z^{NH_3^* \cdots (O^*)} q_{R_x}^{NH_3^* \cdots (O^*)} q_{R_y}^{NH_3^* \cdots (O^*)} q_{xy\varphi}^{NH_3^* \cdots (O^*)}}. \qquad \text{(S13)}$$

The degrees of freedom associated with in-plane motion (x,y) and the C_3-axis rotation (φ) are coupled for NH_3 on O-covered Pt(111). Using the PES from Eq. S1 and S5 we construct the ratio of NH_3 translational partition functions from discrete energy points as those shown in Fig. 4 of the main text. For NH_3 at Pt(111) the C_3-axis rotation is free and can be described by a 1D free rotational partition function (frot):

$$Q_{\mathrm{frot}} = \sqrt{\frac{\pi\, k_B T}{\sigma^2 C}}, \qquad \text{(S14)}$$

where C is the rotational constant associated with the C_3-axis and σ as the symmetry number.

For NH_3 rotation at $p(2 \times 2)$ O/Pt(111) we have to consider that the rotational landscape depends on the in-plane coordinates of NH_3. This means that NH_3 is a hindered rotator which has to be coupled to the in-plane coordinates. The $xy\varphi$ contribution to NH_3 partition function is given by:

$$\frac{q_{xy\varphi}^{NH_3^* \cdots ()}}{q_{xy\varphi}^{NH_3^* \cdots (O^*)}} = \frac{\sum_i \sum_j g_{ij} \exp\left(-\frac{V_{\mathrm{NH3-Pt}}^{\mathrm{DFT}}(x_i, y_j; \{\ldots\}_{\mathrm{rlx}})}{k_B T}\right) Q_{\mathrm{frot}}}{\sum_i \sum_j g_{ij} \exp\left(-\frac{V_{\mathrm{NH3-O/Pt}}(x_i, y_j; \varphi_{\mathrm{rlx}}, \{\ldots\}_{\mathrm{rlx}})}{k_B T}\right) Q_{\mathrm{hrot}}(x_i, y_j)}. \qquad \text{(S15)}$$

The parameter g_{ij} is the fractional contribution of an energy point to the elementary cell in which the energy configurations are sampled. The used configurations are shown in Fig. 4. For example $g_{ij} = 1/6$ at the sharp corner of the grid and $1/2$ at the edges. The index *rlx* indicates that

coordinates were optimized to minimum energy in order to determine the energy of the configuration. The hindered rotational partition function $Q_{\mathrm{hrot}}(x_i, y_j)$ is determined from:

$$Q_{\mathrm{hrot}}(x,y) = \frac{1}{\sigma}\sum_i \exp\left(-\frac{E_i(x,y)}{k_{\mathrm{B}}T}\right), \tag{S16}$$

with $E_i(x,y)$ as the rotational eigenstates of the corresponding rotational potential $V(\varphi;x,y)$ at given in-plane coordinates. The eigenstate energies are derived from numerically solving the 1D nuclear Schrödinger equation as described previously[15].

The frequencies used to characterize the harmonic oscillator partition functions are based on calculations from Offermans *et al.* with some corrections employed. First, we scale all the calculated frequencies of O-atoms (x, y and z) and NH_3's hindered rotation (R_x, R_y) by a factor of 1.05. We find that this scaling yields best agreement with the experimental vibrational spectrum of O* and un-complexed NH_3* at clean Pt(111)[16-17]. The ammonia-surface stretch frequency (z) on clean Pt(111) was determined in previous experiments[17]. Then, the corresponding *harmonic* frequency inside the NH_3-O complex is estimated based on the scaling expected from the Morse-Oscillator with a binding energy increased by the experimental complexation energy.

We also consider the calculated frequencies of the NH_3-O complex to be linked to the calculated complexation energy of $\Delta E_{\mathrm{c}}^{\mathrm{th}} = 0.17$ eV [12] but the experimental complexation energies can be different and this situation requires a correction. Since the frequency shifts inside the complex have relatively small changes (30 to 65 cm^{-1}) we assume that they can be linearly scaled with the complexation energy. The correction scheme was employed to hindered rotational frequencies (R_x and R_y) is given by:

$$\nu_{\mathrm{c}}^{\mathrm{exp}}(\Delta E_{\mathrm{c}}^{\mathrm{NC-AIM}}) \approx \left(\nu^{\mathrm{th}} - \frac{\nu^{\mathrm{th}} - \nu_{\mathrm{c}}^{\mathrm{th}}}{\Delta E_{\mathrm{c}}^{\mathrm{th}}}\Delta E_{\mathrm{c}}^{\mathrm{NC-AIM}}\right) \times 1.05. \tag{S17}$$

The index c indicates the frequency and stabilization energy inside the complex and the superscript indicates the experimental (exp) or theoretical (th) origin of the parameter. Here $\Delta E_{\mathrm{c}}^{\mathrm{NC-AIM}}$ is the complexation energy predicted by NC-AIM. Table S3 lists the frequencies employed to characterize the partition functions.

Table S2: Frequencies of NH_3, O and NH_3-O complex on Pt(111) used for partition function evaluation for optimized NC-AIM with $\Delta E_c^{NC-AIM} = 147\ meV$.

Frequency ↓ \ species →	NH_3	O	NH_3-O
z/cm^{-1}	350.0	-	372.1
R_x/cm^{-1}	678.3	-	715.9
R_y/cm^{-1}	669.9	-	708.4
x/cm^{-1}	-	399.0	339.5

y/cm^{-1}	-	399.0	339.5
z/cm^{-1}	-	483.0	455.5

S3. Modelling of hopping rate constant and diffusion coefficient

S3.1. Relationship between hopping and diffusion

To convert the hopping rate constant to a 2D diffusion coefficient D the jump length l and the average lifetime of a particle at the stable binding site τ are required:

$$D = \frac{l^2}{4\tau}. \tag{S18}$$

In the case of NH_3 on clean Pt(111) the jump length is the Pt-Pt distance a (=2.77 Å, see Fig. S1), while from each binding site the molecule can hop to six neighboring binding sites. Therefore, the average lifetime the molecule spends at the stable binding site is given by:

$$\tau^{\mathrm{Pt(111)}} = \frac{1}{6k_h^{\mathrm{Pt(111)}}}. \tag{S19}$$

The corresponding diffusion coefficient is:

$$D^{\mathrm{Pt(111)}} = \frac{3a^2}{2}k_h^{\mathrm{Pt(111)}}. \tag{S20}$$

On the O-covered Pt(111) the situation is not so straightforward at the first glance. Here, the minimum energy pathway leading from the most stable binding site to another involves a local minimum region—see Fig. 4 (b). This involves two hopping rate constants, which are hopping from binding site 1 (BS_1) to the region around binding site 4 (BS_4)— $k_{\mathrm{h},14}$—and from BS_4 to BS_1—$k_{\mathrm{h},41}$. Justified by the small energy differences between binding site 3 and 4 we make the approximation that these sites form one uniform minimum. Since $k_{\mathrm{h},14}$ is associated with a barrier of 1.1 eV, while $k_{\mathrm{h},41}$ barrier amounts only to ~0.35 eV, the rate determining step for a successful site-to-site exchange involves the hopping from BS_1 to BS_4. Once the molecule has successfully hopped to BS_4 it may hop back to BS_1, where it initially started. This process, absent on clean Pt(111), influences the average lifetime of NH_3 at BS_1 and hence impacts the macroscopic diffusion rate. To understand the relationship between the individual hopping events and the

macroscopic diffusion constant we construct a simple kinetic scheme for NH_3 diffusion on $p(2 \times 2)$ $O/Pt(111)$, shown in Fig. S3.

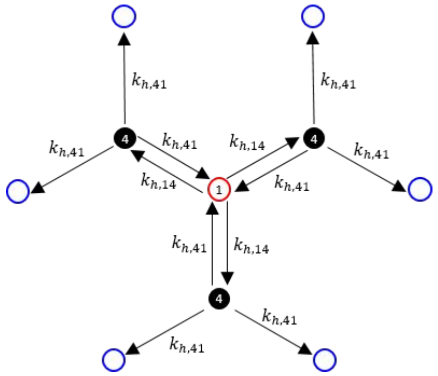

Figure S3: Kinetic scheme to convert site-to-site hopping rate constant on $p(2 \times 2)$ $O/Pt(111)$ to macroscopic diffusion coefficient.

Following the scheme shown in Fig. S3 we can formulate the rate equations of molecules at BS_1 and BS_4 with the aim to understand how τ is related to $k_{h,14}$ and $k_{h,41}$. The rate equations are:

$$[\dot{BS_1}] = -3k_{h,14}[BS_1] + 3k_{h,41}[BS_4] \quad (S21)$$

and

$$[\dot{BS_4}] = 3k_{h,14}[BS_1] - 9k_{h,41}[BS_4]. \quad (S22)$$

Due to the high hopping rate constant $k_{h,41}$ compared to $k_{h,14}$ we can use the steady-state approximation ($[\dot{BS_4}] \approx 0$) for the concentration of molecules at BS_4. This yields:

$$[BS_4]_{SS} = \frac{k_{h,14}}{3k_{h,41}}[BS_1]. \quad (S23)$$

Substituting Eq. S23 to Eq. S21 yields:

$$[\dot{BS_1}] = -2k_{h,14}[BS_1], \quad (S24)$$

from which it becomes clear that the average lifetime of NH_3 at its most stable binding site (BS_1) on $p(2 \times 2)$ $O/Pt(111)$ is

$$\tau^{p(2\times 2)\ \mathrm{O/Pt(111)}} = \frac{1}{2k_{h,14}}. \tag{S25}$$

Considering that on $p(2\times 2)\ \mathrm{O/Pt(111)}$ the jump length l is twice the jump length on pristine Pt(111), we obtain a diffusion coefficient for $p(2\times 2)\ \mathrm{O/Pt(111)}$:

$$D^{p(2\times 2)\ \mathrm{O/Pt(111)}} = 2a^2 k_{h,14}. \tag{S26}$$

The hopping rate constant reported in Table 1 of the main text is $k_h^{p(2\times 2)\ \mathrm{O/Pt(111)}} = k_{h,14}/3$ and reflects the total BS_1-to-BS_1 exchange rate. The evaluation of the hopping rate constant $k_{h,14}$ is explained in Sec. S3.2.

S3.2. Determination of $k_h^{p(2\times 2)\ \mathrm{O/Pt(111)}}$ with harmonic Transition State Theory

We use harmonic Transition State Theory to characterize the hopping rate constant $k_{h,14}$ of NH_3 on $p(2\times 2)\ \mathrm{O/Pt(111)}$:

$$k_{h,14}(T) = \frac{k_\mathrm{B} T Q^{\neq}_{\mathrm{BS}_2}}{h\ \ Q_{\mathrm{BS}_1}} \exp\left(-\frac{W_x^{p(2\times 2)\ \mathrm{O/Pt(111)}}}{k_B T}\right). \quad \text{(S27)}$$

Here Q_{BS_1} is the partition function of NH_3 bound at the BS_1. The BS_2 is the transition state for hopping with $Q^{\neq}_{\mathrm{BS}_2}$ as the corresponding partition function. $W_x^{p(2\times 2)\ \mathrm{O/Pt(111)}}$ is the diffusion barrier derived from NC-AIM fit—see Table 1 in the main text. All degrees of freedom are treated within the harmonic approximation, with exception to C_3 axis rotation of NH_3. This rotation was characterized as a hindered rotor[2] for BS_1 and free rotation at BS_2. The frequencies for the partition functions are obtained with the RPBE-D3 functional since it has overall the best agreement with the experiment—compare Table 1. From $k_{h,14}$ we determine $k_h^{p(2\times 2)\ \mathrm{O/Pt(111)}}$ and $D^{p(2\times 2)\ \mathrm{O/Pt(111)}}$—see Sec. S3.1. Both temperature dependent parameters are reported via the extended Arrhenius equations in Table 1 of the main text.

S4. Additional References

1. Borodin, D.; Rahinov, I.; Galparsoro, O.; Fingerhut, J.; Schwarzer, M.; Golibrzuch, K.; Skoulatakis, G.; Auerbach, D. J.; Kandratsenka, A.; Schwarzer, D.; Kitsopoulos, T. N.; Wodtke, A. M., Kinetics of NH3 Desorption and Diffusion on Pt: Implications for the Ostwald Process. *J Am Chem Soc* **2021,** *143* (43), 18305-18316.

2. Sprowl, L. H.; Campbell, C. T.; Arnadottir, L., Hindered Translator and Hindered Rotor Models for Adsorbates: Partition Functions and Entropies. *J Phys Chem C* **2016,** *120* (18), 9719-9731.

3. Linstrom, P. J.; Mallard, W. G., *NIST Chemistry WebBook, NIST Standard Reference Database Number 69*. National Institute of Standards and Technology: 2022.

4. Derry, G. N.; Ross, P. N., A Work Function Change Study of Oxygen-Adsorption on Pt(111) and Pt(100). *J Chem Phys* **1985,** *82* (6), 2772-2778.

5. Fisher, G. B., The Electronic-Structure of Two Forms of Molecular Ammonia Adsorbed on Pt(111). *Chem Phys Lett* **1981,** *79* (3), 452-458.

6. Jing, A.; Szalewicz, K.; van der Avoird, A., Ammonia dimer: extremely fluxional but still hydrogen bonded. *Nat Commun* **2022,** *13* (1).

7. Su, G. R.; Yang, S.; Jiang, Y. D.; Li, J. T.; Li, S.; Ren, J. C.; Liu, W., Modeling chemical reactions on surfaces: The roles of chemical bonding and van der Waals interactions. *Prog Surf Sci* **2019,** *94* (4).

8. London, F., The General Theory of Molecular Forces. *Trans. Faraday Soc.* **1937,** *33*, 8-26.

9. Paschek, D.; Geiger, A., Molecular dynamics simulations of ammonia adsorbed on titanium dioxide (rutile) surfaces. *AIP Conference Proceedings* **1995,** *330* (1), 349-355.

10. Bondi, A., Van Der Waals Volumes and Radii. *J Phys Chem-Us* **1964,** *68* (3), 441-451.

11. Borodin, D.; Hertl, N.; Park, G. B.; Schwarzer, M.; Fingerhut, J.; Wang, Y. Q.; Zuo, J. X.; Nitz, F.; Skoulatakis, G.; Kandratsenka, A.; Auerbach, D. J.; Schwarzer, D.; Guo, H.; Kitsopoulos, T. N.; Wodtke, A. M., Quantum effects in thermal reaction rates at metal surfaces. *Science* **2022,** *377* (6604), 394-398.

12. Offermans, W. K.; Jansen, A. P. J.; van Santen, R. A., Ammonia activation on platinum {111}: A density functional theory study. *Surf Sci* **2006,** *600* (9), 1714-1734.

13. Borodin, D.; Rahinov, I.; Fingerhut, J.; Schwarzer, M.; Hörandl, S.; Skoulatakis, G.; Schwarzer, D.; Kitsopoulos, T. N.; Wodtke, A. M., NO Binding Energies to and Diffusion Barrier on Pd Obtained with Velocity-Resolved Kinetics. *J Phys Chem C* **2021,** *125* (21), 11773-11781.

14. Borodin, D.; Rahinov, I.; Shirhatti, P. R.; Huang, M.; Kandratsenka, A.; Auerbach, D. J.; Zhong, T.; Guo, H.; Schwarzer, D.; Kitsopoulos, T. N.; Wodtke, A. M., Following the microscopic pathway to adsorption through chemisorption and physisorption wells. *Science* **2020,** *369* (6510), 1461-1465.

15. McClurg, R. B.; Flagan, R. C.; Goddard, W. A., The hindered rotor density-of-states interpolation function. *J Chem Phys* **1997,** *106* (16), 6675-6680.

16. Gland, J. L.; Sexton, B. A.; Fisher, G. B., Oxygen Interactions with the Pt(111) Surface. *Surf Sci* **1980,** *95* (2-3), 587-602.

17. Sexton, B. A.; Mitchell, G. E., Vibrational-Spectra of Ammonia Chemisorbed on Platinum (111) .1. Identification of Chemisorbed States. *Surf Sci* **1980,** *99* (3), 523-538.
